# Emergence of an XDR *Klebsiella pneumoniae* ST5491 strain co-harboring NDM-5, MCR-1.1, tmexCD1-toprJ1, and a novel plasmid carrying CTX-M-15

**DOI:** 10.3389/fmicb.2025.1581851

**Published:** 2025-04-30

**Authors:** Yinfei Fang, Xiangchen Li, Zhaoxia Wu, Yongjin Fang, Yeping Wang, Xiaobing Li, Lihong Bu, Keqiang Chen, Kai Shen, Yongjun Ma, Mingjuan Wu

**Affiliations:** ^1^Department of Clinical Laboratory, Affiliated Jinhua Hospital, Zhejiang University School of Medicine, Jinhua, China; ^2^Jiaxing Key Laboratory of Clinical Laboratory Diagnostics and Translational Research, Affiliated Hospital of Jiaxing University, Jiaxing, China; ^3^Department of Otolaryngology, Affiliated Jinhua Hospital, Zhejiang University School of Medicine, Jinhua, China; ^4^Department of Pediatrics, Jinhua Women’s and Children’s Hospital, Jinhua, China; ^5^Department of Health Management, Affiliated Jinhua Hospital, Zhejiang University School of Medicine, Jinhua, China

**Keywords:** *Klebsiella pneumoniae*, extensively drug-resistant (XDR), genomic analysis, antimicrobial resistance (AMR), IncFIA/IncFII/IncQ1 hybrid plasmid

## Abstract

**Objective:**

The rapid emergence of antimicrobial resistance (AMR) in *Klebsiella pneumoniae* poses a significant global health threat. The study aimed to analyze and describe the genomic architecture and resistance mechanisms of an extensively drug-resistant (XDR) *K. pneumoniae* isolate, KP09, by focusing on plasmids that harbor multiple resistance genes, including *tmexCD1-toprJ1*, *bla_CTX-M-15_*, *bla_NDM-5_*, and *mcr-1.1*.

**Methods:**

The KP09 strain, isolated from a clinical sample, was subjected to antimicrobial susceptibility testing and conjugation experiments. Whole-genome sequencing with both long- and short-read methods facilitated hybrid assembly for complete genome reconstruction. Bioinformatics analyses identified resistance genes, plasmid structures, and sequence types (STs), whereas comparative genomic analysis elucidated the context and dissemination mechanisms of resistance determinants.

**Results:**

KP09 exhibited broad-spectrum resistance to carbapenems, colistin, eravacycline, and tigecycline, and only remained susceptible to cefiderocol. The conjugation experiments successfully produced four transconjugants, each carrying specific plasmids: JKP09-1 harbored the *tmexCD1-toprJ1* gene, JKP09-2 harbored *tmexCD1-toprJ1* and *mcr-1.1* genes, JKP09-3 harbored the *mcr-1.1* gene, and JKP09-4 harbored *bla_NDM-5_* and *mcr-1.1* genes. Genomic analysis revealed a novel IncFIA/IncFII/IncQ1 hybrid plasmid carrying *bla*_CTX-M-15_, along with a large conjugative plasmid encoding the tmexCD1-toprJ1 efflux pump. The *bla*_NDM-5_ and *mcr-1.1* genes were located in separate IncX-type plasmids, suggesting independent dissemination pathways. Furthermore, KP09 was identified as a new sequence type, ST5491, closely related to the endemic ST15 clone. The comparative analysis highlighted the role of mobile genetic elements, such as IS*26* and IS*Ecp1*, in facilitating the spread of resistance genes.

**Conclusion:**

This study provides critical information on the genetic mechanisms that drive AMR in *K. pneumoniae*, including the identification of a novel *bla*_CTX-M-15_ encoding IncFIA/IncFII/IncQ1 hybrid plasmid and the emergence of the ST5491 strain. Understanding the genetic basis of resistance is essential to inform public health interventions and mitigate the impact of AMR.

## 1 Introduction

*Klebsiella pneumoniae* is a common Gram-negative opportunistic pathogen and the most clinically significant species within the *Klebsiella* genus (Bengoechea and Sa Pessoa, 2019). It is associated with various infections, including urinary tract infections, pneumonia, bacteremia, and liver abscesses. Historically, antibiotics such as β-lactams and aminoglycosides have been effective treatments; however, overuse and misuse of these drugs have led to the rapid emergence of antimicrobial resistance (AMR), complicating clinical management (Riwu et al., 2020). In particular, data from China’s national surveillance program, CHINET, revealed a striking increase in imipenem and meropenem resistance rates in *K. pneumoniae*, increasing from 2.9% in 2005 to 30.0% in 2023 (Pan et al., 2024).

A major driver of AMR in *K. pneumoniae* is the dissemination of extended-spectrum β-lactamases (ESBLs), particularly the CTX-M type, such as CTX-M-15, which confers resistance to β-lactam antibiotics like penicillins and cephalosporins (Riwu et al., 2020). These resistance genes, often plasmid-borne (e.g., *bla*_CTX-M_), facilitate horizontal gene transfer among bacterial populations (Smiline et al., 2018). Carbapenems, once considered the last line of defense against multidrug-resistant (MDR) bacteria, are now compromised by carbapenemase-producing strains harboring *bla*_NDM_ genes encoding New Delhi metallo-β-lactamase (NDM) (Acman et al., 2022). Colistin, a last resort treatment for Gram-negative infections, has been undermined by the emergence of *mcr* genes, especially *mcr-1*, first identified in China in 2015 (Liu et al., 2024). Compounding these challenges, resistance to tigecycline, a key therapy for extensively drug-resistant (XDR) *K. pneumoniae*, is increasing due to ribosomal mutations (e.g., *rpsJ*) and plasmid-mediated genes [e.g., *tet(X)* variants, *tet(A)*, *tet(M)*] (Sheng et al., 2014). Of particular concern is the plasmid-encoded RND efflux pump *tmexCD1-toprJ1*, first identified in *K. pneumoniae* in China in 2020, which has rapidly disseminated in both agricultural and nosocomial settings (Lv et al., 2020).

The growing prevalence of MDR, XDR, and even pandrug-resistant (PDR) *K. pneumoniae* underscores the urgency of addressing AMR (Navon-Venezia et al., 2017). These strains, resistant to most or all available antibiotics, present significant challenges for clinical treatment and infection control. Furthermore, the emergence of novel sequence types (STs) associated with specific resistance genes has reshaped the epidemiological landscape, with implications for both the spread of AMR and patient outcomes (Wang et al., 2012; Peirano et al., 2020). Characterizing these STs and their genetic determinants is crucial to understanding AMR dissemination and informing strategies to mitigate its impact.

In this study, we performed a comprehensive genomic analysis of a clinical isolate of XDR *K. pneumoniae*, KP09, using long- and short-read sequencing. Our investigation focused on the structural characterization of plasmids that harbor multiple antimicrobial resistance genes (*tmexCD1-toprJ1*, *bla*_CTX-M-15_, *bla*_NDM-5_, and *mcr-1.1*). By comparing these plasmids with previously reported sequences, we sought to elucidate their genetic architecture and the mechanisms underlying the dissemination of AMR genes. This study aims to improve our understanding of AMR in *K. pneumoniae* and provides valuable information to guide the development of strategies to combat this global health threat.

## 2 Materials and methods

### 2.1 Bacterial isolation and identification

The *K. pneumoniae* strain KP09 was isolated from an intestinal sample of a 45-year-old woman at the Affiliated Jinhua Hospital, Zhejiang University School of Medicine in Zhejiang Province, on 8 September 2022, as part of the carbapenem resistance surveillance program. The patient was admitted to the Department of Nephrology with a diagnosis of impaired kidney function and no antibiotics were administered during hospitalization. The strain KP09 was identified by matrix-assisted laser desorption ionization time of flight mass spectrometry (MALDI-TOF MS; Microflex LH/ST, Bruker Daltonik GmbH, Bremen, Germany).

### 2.2 Antimicrobial susceptibility testing

The antimicrobial susceptibility of the strain to eravacycline and cefiderocol was determined using the E test method (E-test Liofilchem S.r.l., Italy; MH Comagar (Shanghai) Co., Ltd., China), whereas susceptibilities to other antibiotics were evaluated using the broth microdilution method (Kangtai Bio Co., Ltd., China), interpreted according to the Institute of Clinical and Laboratory Standards (CLSI) of 2024. Resistance break points for imipenem, meropenem, ertapenem, ceftazidime, cefotaxime, cefepime, cefiderocol, aztreonam, piperacillin/tazobactam, ceftazidime/avibactam, levofloxacin, ciprofloxacin, and amikacin were determined according to CLSI guidelines. For cefoperazone/sulbactam, the breakpoint was established based on the cefoperazone standard. Resistance breakpoints for colistin and tigecycline were interpreted according to the guidelines of the European Committee on Antimicrobial Susceptibility Testing (EUCAST) v.12.0. The resistance breakpoint for eravacycline was interpreted according to the guidelines of the Expert Committee of the National Antimicrobial Susceptibility Testing Commission (ChinaCAST).

### 2.3 Antimicrobial synergy test

The *in vitro* antibacterial activity of eravacycline combined with ceftazidime/avibactam, levofloxacin, colistin, and sulbactam against KP09 was detected using the checkerboard method. FIC (Fractional Inhibitory Concentration) is used to assess the effect of two antimicrobial drug interactions. FIC = (MIC of drug A in combination)/(MIC of drug A alone) + (MIC of drug B in combination)/(MIC of drug B alone). FIC ≤ 0.5 indicates a synergistic effect of two drugs, 0.5 < FICI ≤ 1 indicates an additive effect of two drugs, 1 < FICI ≤ 2 indicates no interaction between the two drugs, FIC > 2 indicating antagonism between the two drugs.

### 2.4 Conjugation experiments

Conjugation experiments were performed using KP09 as the donor strain and sodium azide resistant *E. coli* J53 as the recipient strain. The conjugation method was performed as previously described (Pan et al., 2024). Briefly, donor and recipient strains were cultured 4 h in Luria-Bertani broth (LB) (Sangon Biotech, Shanghai, China) at 37°C with shaking at 200 rpm. Equal volumes of donor and recipient cultures were mixed at a 1:1 ratio and then filtered through a 0.22 μm membrane filter placed on moistened commercial MH agar plates [Comagar (Shanghai) Co., Ltd., China]. The mixture was incubated at 37°C for 18 h. After incubation, bacteria were resuspended in LB broth and selected on Mueller–Hinton (MH) agar (Oxoid, Hampshire, United Kingdom) supplemented with:

100 μg/mL sodium azide and 1 μg/mL tigecycline for tmexCD1-toprJ1.
100 μg/mL sodium azide and 1 μg/mL colistin for MCR-1.1.
100 μg/mL sodium azide and 1 μg/mL meropenem for NDM-5.

Conjugation experiments were performed in three parallel replicates. The presumptive conjugants were confirmed by MALDI-TOF MS and PCR (T100TM) (Thermal Cycler, Bio-RAD, United States) with specific primers (primer sequences were provided in Supplementary Table S1). The conjugation efficiency was calculated by dividing the number of transconjugants by the number of donor strains. The MICs of the selected transconjugants were determined using the method described above.

### 2.5 Whole-genome sequencing

Genomic DNA was extracted using the Wizard^®^ Genomic DNA Purification Kit (Promega, Madison, WI) following the manufacturer’s protocol. Whole genome sequencing of KP09 was carried out using both short read Illumina NovaSeq 6000 (Illumina Inc., San Diego, CA, United States) and long read Oxford Nanopore MinION (Oxford Nanopore Technologies, Oxford, United Kingdom) platforms, according to the manufacturer’s instructions.

### 2.6 Bioinformatics analysis

The long and short WGS reads were trimmed using Filtlong^1^ and fastp, respectively (Chen et al., 2018). Hybrid assembly was conducted with Unicycler v0.5.0, using default settings, resulting in a complete genome assembly (Wick et al., 2017). Additionally, we performed genome assembly using the long-read assembler Flye with default parameters to verify the completeness of chromosome and plasmid assembly by Unicycler (Kolmogorov et al., 2019). The quality of the genome assembly was evaluated using QUAST v5.0.2, CheckM v1.1.3, and fastANI v1.34, with the *K. pneumoniae* genome HS11286 (NCBI Assembly ID: ASM24018v2) as a reference (Gurevich et al., 2013; Parks et al., 2015; Musiał et al., 2024).

WGS-based *Klebsiella* serotyping was performed with Kleborate with default settings (Lam et al., 2021). Sequences obtained were submitted to the PubMLST database^2^ to determine allele numbers and specific sequence types (ST). BacWGSTdb 2.0 was used to perform the core genome multilocus sequence typing (cgMLST) analysis with other closely related *K. pneumoniae* isolates deposited in the NCBI GenBank database (Feng et al., 2021). The closest relatives were identified using the Similar Genome Finder tool in the BV-BRC database, which uses the Mash/MinHash algorithm (v.2.3) for comparative genomic analysis (Olson et al., 2023).

Core genome alignment and single nucleotide polymorphism (cgSNP) calling were performed using snippy v.4.6.0.^3^ Subsequently, phylogenetic reconstruction was performed using RAxML v.8.2.9 based on the alignment of the cgSNP, following the removal of predicted recombination sites through Gubbins v.2.1.0 (Stamatakis, 2014; Croucher et al., 2015). The resulting phylogenetic tree, integrated with relevant metadata, was visualized and annotated using the interactive Tree of Life (iTOL) web application (Letunic and Bork, 2021).

MOB suite v.3.1.4 was used to predict plasmid sequences from the assembled genome and to identify their replicon types, mobility, and host range (Robertson and Nash, 2018). Gene predictions and functional annotations were performed using the RAST server (Overbeek et al., 2014). The presence of acquired antibiotic resistance genes (ARGs) and chromosomal resistance mutations was detected with ResFinder v4.5.0 (Florensa et al., 2022). Virulence factors (VFs) were screened using the VFDB database (Liu et al., 2022). The search for insertion sequence (IS) elements and their characterization down to the family level was conducted using digIS and ISfinder (Siguier et al., 2006; Puterová and Martínek, 2021). IntegronFinder v.2.0 was used to detect complete integrons (Néron et al., 2022). Plasmid alignment was generated using BRIG v.0.95 (Alikhan et al., 2011).

## 3 Results

### 3.1 Phenotype and genotype of *Klebsiella pneumoniae* KP09

Antibiotic susceptibility testing (AST) results showed that KP09 exhibited resistance to nearly all antibiotics tested, including cephalosporin, carbapenem, aminoglycoside, fluoroquinolone, penicillin (β-lactamase inhibitor) classes, tigecycline, eravacycline, and colistin, reflecting a broad spectrum of antimicrobial resistance, and only remained susceptible to cefiderocol (Table 1).

**Table 1:** Antibiotic susceptibilities of *K. pneumoniae* KP09, transconjugant JKP09-1, JKP09-2, JKP09-3, and JKP09-4.

Antimicrobial susceptibility	*K. pneumoniae* KP09	Transconjugant JKP09-1 (tmexCD1-toprJ1)	Transconjugant JKP09-2 (tmexCD1-toprJ1 + MCR-1.1)	Transconjugant JKP09-3 (MCR-1.1)	Transconjugant JKP09-4 (NDM-5 + MCR-1.1)	*E. coli* J53
	MIC (μg/mL)					
Amikacin	>128 (R)	>128 (R)	>128 (R)	<4 (S)	<4 (S)	<4 (S)
Aztreonam	>128 (R)	<4 (S)	<4 (S)	<4 (S)	<4 (S)	<4 (S)
Piperacillin/Tazobactam	>256/4 (R)	<8/4 (S)	<8/4 (S)	<8/4 (S)	128/4 (R)	<8/4 (S)
Cefmetazole	128 (R)	8 (S)	4 (S)	<2 (S)	16 (S)	<2 (S)
Cefotaxime	>128 (R)	<4 (S)	<4 (S)	<4 (S)	128 (R)	<4 (S)
Ceftazidime	>128 (R)	<2 (S)	<2 (S)	<2 (S)	>128 (R)	2 (S)
Cefepime	>64 (R)	<4 (S)	<4 (S)	<4 (S)	16 (R)	4 (S)
Cefoperazone/Sulbactam	>256/128 (R)	<8/4 (S)	<8/4 (S)	<8/4 (S)	128/64 (R)	<8/4 (S)
Ceftazidime/Avibactam	>64/4 (R)	<0.5/4 (S)	<0.5/4 (S)	<0.5/4 (S)	>64/4 (R)	<0.5/4 (S)
Ciprofloxacin	>32 (R)	<1 (S)	<1 (S)	<1 (S)	<1 (S)	<1 (S)
Ertapenem	128 (R)	<0.008 (S)	<0.008 (S)	<0.008 (S)	4 (R)	<0.008 (S)
Imipenem	32 (R)	0.25 (S)	0.25 (S)	0.25 (S)	4 (R)	0.25 (S)
Meropenem	64 (R)	<0.016 (S)	<0.016 (S)	<0.016 (S)	4 (R)	<0.016 (S)
Colistin	8 (R)	0.5 (S)	8 (R)	4 (R)	4 (R)	0.5 (S)
Tigecycline	4 (R)	2 (R)	2 (R)	0.25 (S)	0.25 (S)	0.25 (S)
Eravacycline	8 (R)	1.5 (R)	1.5 (R)	0.125 (S)	0.125 (S)	0.125 (S)
Cefiderocol	2 (S)	<0.016 (S)	<0.016 (S)	<0.016 (S)	0.25 (S)	<0.016 (S)

The results of the antimicrobial synergy testing showed that the combination of eravacycline and colistin had a synergistic effect on KP09, eravacycline combined with ceftazidime/avibactam and eravacycline combined with levofloxacin showed an additive effect, whereas eravacycline combined with sulbactam did not exhibit any interaction (Supplementary Table S2).

We successfully obtained four different transconjugants by conjugation experiments, JKP09-1, JKP09-2, JKP09-3, and JKP09-4 (PCR results are provided in Supplementary Figure S1). JKP09-1 and JKP09-2 were isolated from MH Agar with sodium azide and tigecycline, JKP09-1 harbors the *tmexCD1-toprJ1* gene, the MIC for tigecycline is elevated 8 times compared to J53 when the MIC for eravacycline is elevated more than 10 times. The pKP09-tmexCD-toprJ plasmid could be successfully transferred into J53 at a conjugation frequency of 8.3 × 10^−6^. JKP09-2 harbors the *tmexCD1-toprJ1* and *mcr-1.1* gene, compared to JKP09-1, the MIC value of colistin reached 8. JKP09-3 was isolated from MH Agar with sodium azide and colistin, it harbors the *mcr-1.1* gene, the MIC for colistin was 8-fold higher than that of J53. The pKP09-MCR plasmid could be successfully transferred into J53 at a conjugation frequency of 2.1 × 10^−3^. JKP094 was isolated from MH Agar with sodium azide and meropenem, harbored the *bla*_NDM-5_ and *mcr-1.1* gene, and showed significantly increased MIC values for all cephalosporins and carbapenems. The pKP09-NDM plasmid could be successfully transferred into J53 at a conjugation frequency of 2.5 × 10^−5^.

**Figure 1:**
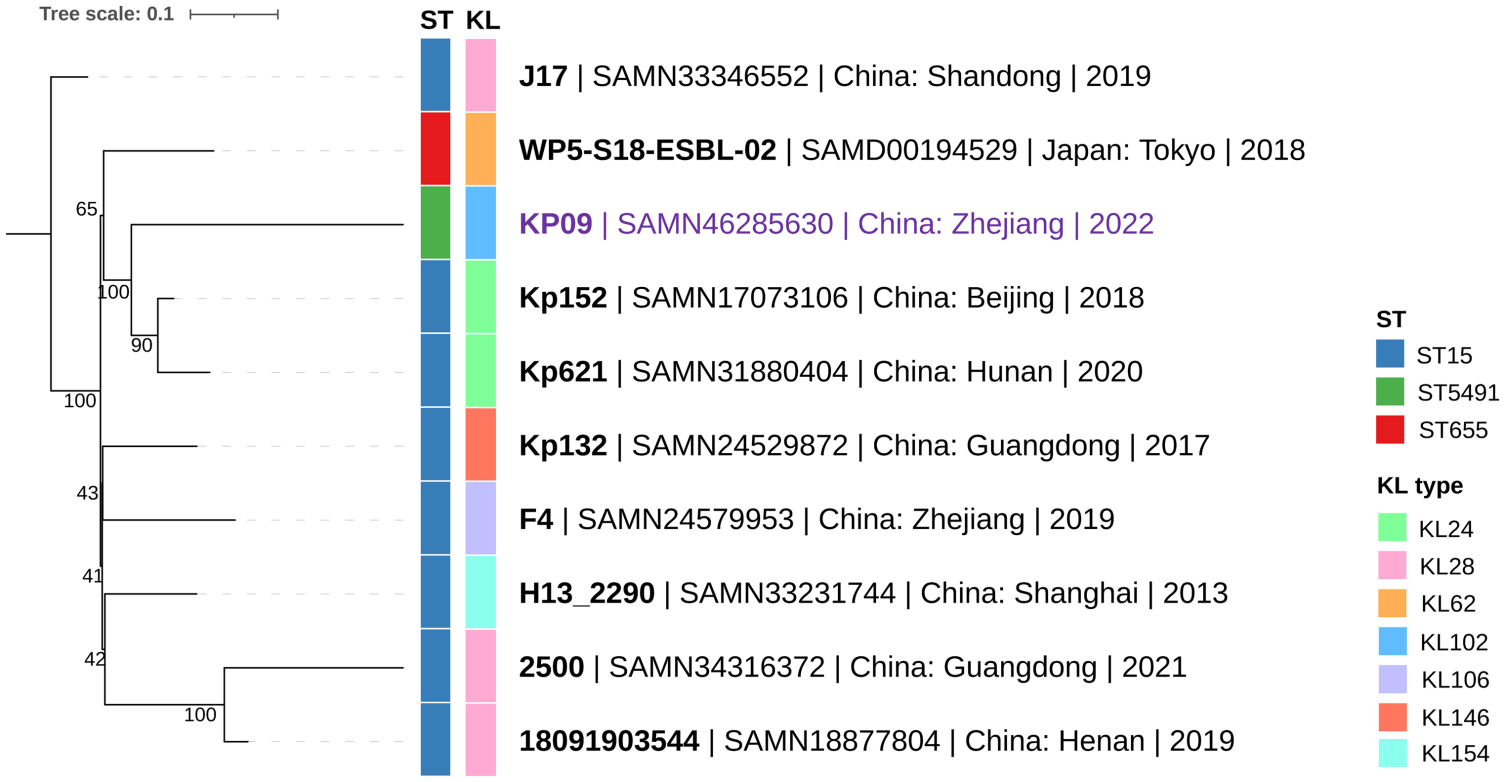
Phylogenetic relationship between KP09 and its nine closely related *K. pneumoniae* strains using the cgSNP strategy. The color bars on the phylogenetic tree represent ST-type and KL (K-locus) from the innermost to the outermost layers. The branch labels are formatted as strain name | NCBI BioSample accession | collection area | collection year.

*In silico* MLST typing classified KP09 as ST5491 (*gapA*-*infB*-*mdh*-*pgi*-*phoE*-*rpoB*-*tonB*: 1-1-1-1-1-1-16), which differs from the endemic ST15 clone (1-1-1-1-1-1-1) by a single *tonB* allele. Kleborate analysis assigned KP09 to the KL102-O1 capsule type. Using the Similar Genome Finder tool in the BV-BRC database, we identified high genomic similarity (mash distance <0.0022) between KP09 and eight ST15 strains isolated in China and one ST655 strain from Japan. cgSNP analysis of these 10 *K. pneumoniae* strains revealed fewer than 200 cgSNP differences. Phylogenetic reconstruction showed that KP09 was most closely related to Kp152 (NCBI BioSample Accession: SAMN17073106), a strain isolated from a dog in Beijing in 2018, with a cgSNP distance of 126 (Figure 1).

### 3.2 Genomic characteristics and antimicrobial resistance genotype analysis

The complete genome assembly achieved using the hybrid assembler Unicycler revealed that strain KP09 consists of one chromosome (5.14 Mb) and nine plasmids (2.01 to 258.64 kb), encoding 5,384 predicted ORFs. The number of contigs was the same as the long-read assembly from Flye (Supplementary Table S3). KP09 has a GC content of 56.6% and contains no gaps or Ns, with an estimated completeness of 98.4% and an average nucleotide identity (ANI) of 99.0% compared with the reference genome of *K. pneumoniae* HS11286. For the five small plasmids (pKP09-5 to pKP09-9) with lengths less than 30 kb, a search was conducted using megaBLAST in NCBI GenBank. For each of these plasmids, at least two homologous plasmids were identified, with a sequence coverage exceeding 95% and an identity greater than 99%. This indicates that these plasmids are indeed present (Supplementary Figure S2).

In total, 41 acquired ARGs were detected, 19 of which were associated with the observed resistance phenotypes (Table 2). In particular, five ARGs were located on the chromosome and other 36 ARGs were harbored by five plasmids. The plasmids pKP09-tmexCD-toprJ, pKP09-CTX-M and pKP09-5 carried 18, 14, and 2 ARGs, respectively, whereas pKP09-MCR and pKP09-NDM each carried one ARG. VFDB led to detection of 104 VFs, but was negative for all yersiniabactin (*ybt*), colibactin (*clb*), and aerobactin (*iuc*).

**Table 2:** Genetic mechanisms of multidrug resistance of *K. pneumoniae* KP09.

Mechanism of resistance	Antibiotics	Relevant genes
Antibiotic inactivation	Aminoglycoside	*APH(3″)-Ib*, *APH(6)-Id*, *APH(3′)-Ia*, *AAC(3)-Iid*, *aadA16*, *AAC(6′)-Ib-cr6*, *aadA2*, *ANT(3″)-Iia*, *AAC(3)-IV*, *APH(4)-Ia*, *armA*
	β-lactam	*bla*_DHA-1_, *bla*_CTX-M-15_, *bla*_NDM-5_
	Macrolide	*mphA*, *mphE*, *msrE*
	Rifamycin	*ARR-3*
Replacement of antibiotic target	Sulfonamide	*sul1*, *sul2*, *sul3*
	Peptide-based	*mcr-1.1*
	Diaminopyrimidine	*dfrA27*
Antibiotic efflux	Tetracycline	*tet(A)*, *tmexCD1-toprJ1*
	Acridine	*qacE∆1*
	Phenicol	*cmlA1*
Antibiotic target protection	Quinolone	*QnrB4*

### 3.3 Comparative analysis of plasmids containing pKP09-tmexCD-toprJ and tmexCD-toprJ

The largest plasmid pKP09-tmexCD-toprJ was a 258.6 kb plasmid with an average G + C content of 46.8%, encoding 151 predicted ORFs. MOB-suite revealed that pKP09-tmexCD-toprJ was a multi-replicon plasmid with IncFIB/IncHI1B-typed replicons, belonging to the plasmid cluster AA405 and was predicted to be conjugative due to the presence of relaxase and mate pair formation markers. This plasmid exhibited the highest similarity (99% coverage and 99% identity) with two *tmexCD*-*toprJ*-harboring IncFIB/IncHI1B-type plasmids (accession: CP097191 and CP110146) from *K. pneumoniae* strains 15–652 and YZ22CK024, respectively. According to the metadata, strain 15–652 was isolated from human urine in Quzhou City, Zhejiang Province, whereas YZ22CK024 was isolated from chicken in Yangzhou City, Jiangsu Province.

A total of 18 different ARGs, including multidrug resistance (MDR) efflux pump gene cluster *tmexCD1-toprJ1*, eight aminoglycoside-related ARGs [*aph(4)-Ia*, *aph(6)-Id*, *aph(3′)-Ia*, *aph(3″)-Ib*, *aadA1*, *aadA2b*, *aac(3)-IV*, and *armA*], one β-lactam-related ARG (*bla*_DHA-1_), one quinolone-related ARG (*QnrB4*), two macrolide-related ARGs (*mphE* and msrE), two sulfonamide-related ARGs (*sul1* and *sul3*) and one phenicol-related ARG (*cmlA1*) were identified. These resistance genes were densely clustered within a 65,340 bp MDR encoding region, with the 5′ end flanked by a Tn*3* family transposase Tn*As1* and the 3′ end by an IS*1* family transposase IS*1R*. The MDR region contained an array of mobile elements including IS*Kpn41*, IS*1*, IS*406*, IS*903*, ISE*c29*, ISE*c35*, ISE*c59*, Tn*5393*, four copies of IS*26* and two copies of Tn*AS1*. Additionally, an IS*406*-linked class 1 integron containing an ARG cassette (*aadA2b*, *cmlA1*, and *aadA1*) was identified within this MDR region.

**Figure 2:**
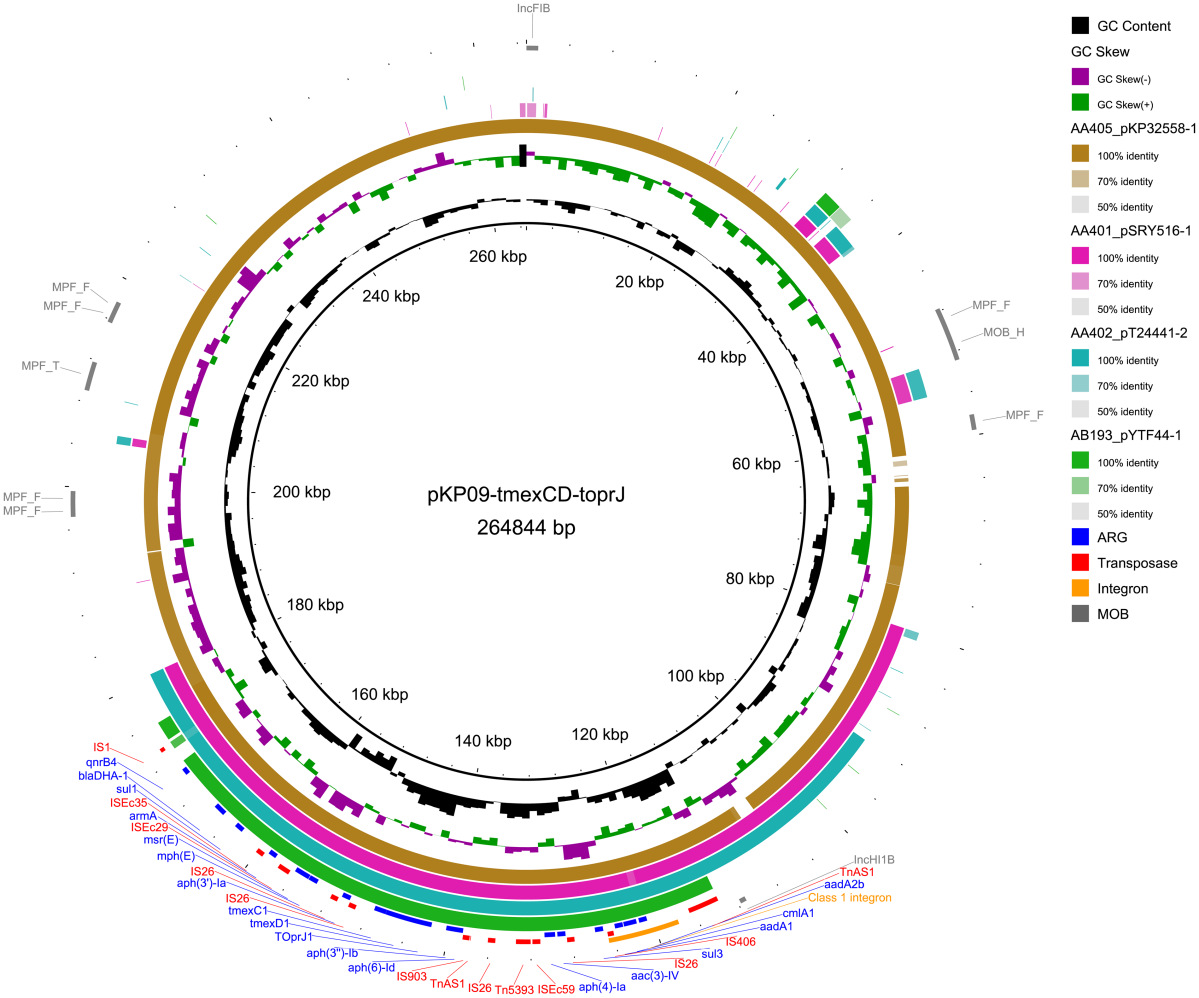
Circular plasmid map of pKP09-tmexCD-toprJ. Concentric rings represent the similarity between the reference sequence (pKP09-tmexCD-toprJ) in the inner ring and the other four plasmids that resemble tmexCD-toprJ in the outer rings. Resistance and transposase genes are marked in blue and red fonts, respectively. The sequences related to integron and mobility are highlighted with orange and gray ring, respectively.

A megaBLAST search of this MDR region in the NCBI database identified 45 plasmid hits from *K. pneumoniae*, *K. oxytoca*, and *E. coli* isolates, each with >90% of sequence identity and coverage. Alongside the IncFIB and IncHI1B replicons, the IncFIA, IncR, IncU, and IncX1 replicons were also present. The MOB typer divided these plasmids into four clusters, with the largest cluster containing pKP09-tmexCD-toprJ and 29 other conjugative plasmids, whereas the remaining clusters comprised nonmobilizable plasmids. A further comparison of four representative plasmids with pKP09-tmexCD-toprJ revealed that this MDR region is highly conserved, suggesting that it was likely acquired by horizontal gene transfer and is transmissible across various plasmid backbones (Figure 2 and Supplementary Table S4).

### 3.4 A novel CTX-M-15-encoding hybrid plasmid

The second-largest plasmid, pKP09-CTX-M, had a total length of 124.5 kb and a GC content of 52.36%. It encoded three types of replication proteins: IncFIA, IncFII, and IncQ1, and was predicted to be conjugative. pKP09-CTX-M harbored 14 different ARGs, including one β-lactam-related ARG (*bla_CTX-M-15_*), six aminoglycoside-related ARGs [*APH(3″)-Ib*, *APH(6)-Id*, *APH(3′)-Ia*, *AAC(3)-IId*, and *aadA16*], two sulfonamide-related ARGs (*sul1* and *sul2*), one tetracycline-related ARG *[tet(A)]*, one macrolide-related ARG (*mphA*), one diaminopyrimidine-related ARG (*dfrA27*), one rifamycin-related ARG (*arr-3*), and one acridine dye-related ARG (*qacE 1*).

**Figure 3:**
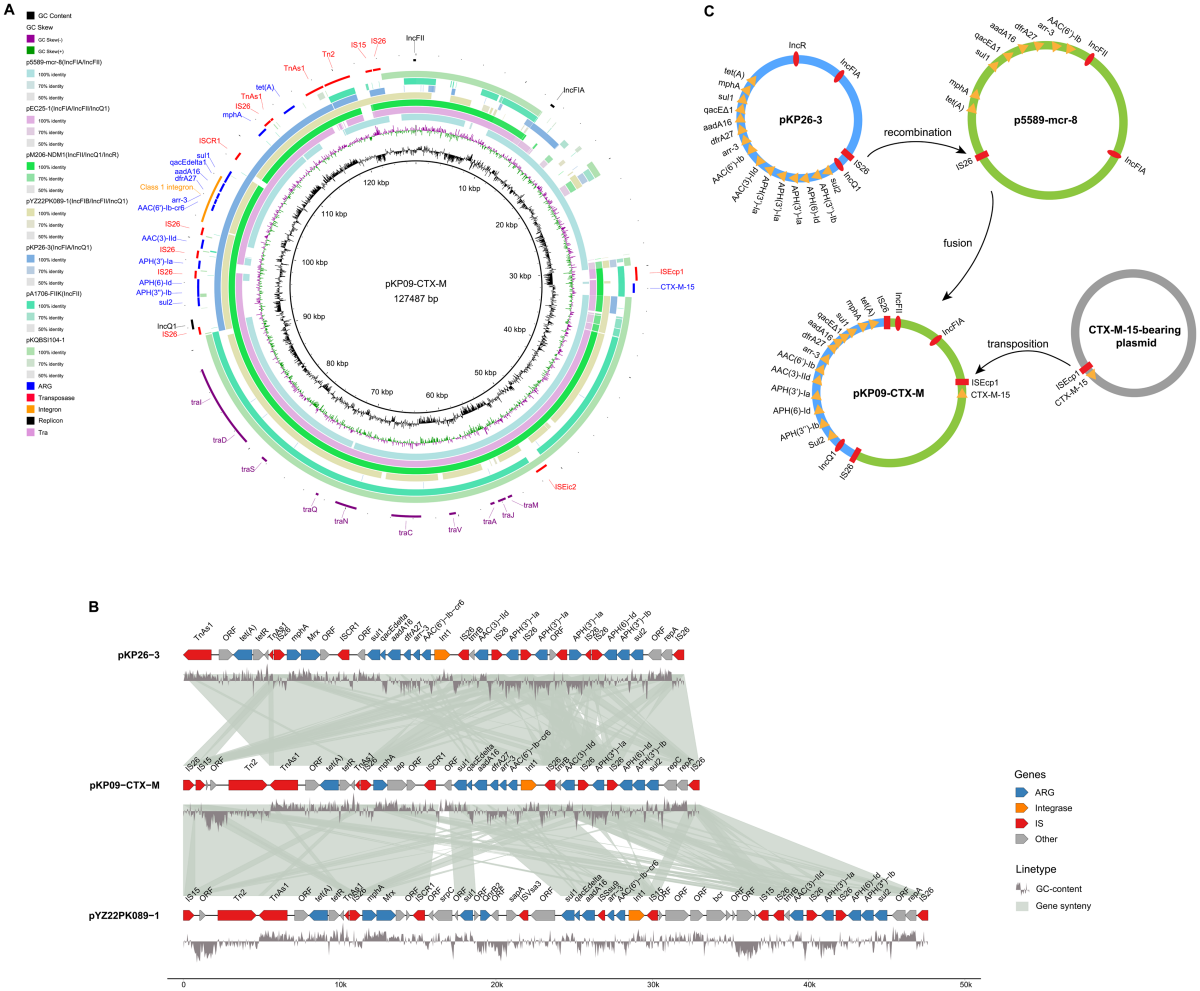
Genetic diagram of the plasmid pKP09-CTX-M. **(A)** The map of plasmids of pKP09-CTX-M and other five plasmids, including p5589-mcr-8, pEC25-1, pM206-NDM1, pYZ22PK089-1, and pKP26-3. **(B)** Comparison of the MDR regions on pKP09-CTX-M, pKP26-3, and pYZ22PK089-1. The shadow represents >95% sequence identity. The GC content is displayed below the MDR region. The figure was built using the R package genomes (https://github.com/thackl/gggenomes). **(C)** Putative mechanisms of plasmid fusion mediated by homologous recombination and transposition.

Based on a megaBLAST search, the plasmid most similar to pKP09-CTX-M was p5589-mcr-8, also derived from *K. pneumoniae*, with 99.7% sequence identity and 84% coverage (Supplementary Table S5). p5589-mcr-8 was a 134.3 kb hybrid plasmid of the IncFIA/IncFII type. Comparison of their ARG compositions revealed that p5589-mcr-8 lacks not only *bla*_CTX-M-15_ but also *AAC(3)-IId*, *APH(6)-Id*, *APH(3″)-Ib*, and *sul2* (Figure 3A). In pKP09-CTX-M, *bla*_CTX-M-15_ was located upstream of a hypothetical protein gene and an IS*Ecp1* transposase gene, both of which are also absent in p5589-mcr-8. The comparison revealed that this 2.3 kb region shared 100% sequence identity and coverage with numerous plasmids carrying *bla*_CTX-M-15_.

All other ARGs in pKP09-CTX-M were tightly clustered within a 32.2 kb MDR region, as well as with several mobile elements, including IS*26*, IS*CR1*, Tn*As1*, Tn*2*, and IS*15* (Figure 3A). This hypervariable MDR region consisted of four IS*26* elements flanking the following three different segments: (i) IS*26-repA-repC-sul2-APH(3″)-Ib-APH(6)-Id-IS26*, (ii) IS*26-APH(3′)-Ia-IS26*, (iii) an ISCR1 linked complex class 1 integron, formed by IS*26-Int1-AAC(6′)--Ib-cr6-arr-3-dfrA27-aadA16-qacE∆1-sul1*-ISC*R1*-mphR (A)-mrx (A) -mphA-IS26, and (iv) IS26-TnAs1-tetR-tet(A)-eamA-TnAs1. A megaBLAST search detected two *K. pneumoniae* plasmid hits, IncFIA/IncQ1-plasmid pKP26-3 and pYZ22PK089-1 with >99.9% identity and ≥95% coverage (Figures 3A,B and Supplementary Table S5). In particular, pKP26-3 and pYZ22PK089-1 were isolated from chicken and pork, respectively, with sampling locations Hangzhou in Zhejiang Province and Yangzhou in Jiangsu Province.

Based on the sequence analysis described above, we propose a model for the fusion and resolution of pKP09-CTX-M, as illustrated in Figure 3C. In this model, IS*26* was positioned upstream of the MDR region, whereas the IncQ1 replicon protein in pKP26-3 recognized and attached the target site duplication (TSD) (AGCTGCAC) in the conjugative plasmid p5589-mcr-8. Subsequently, replicative transposition occurred, resulting in the insertion of the MDR and IncQ1 replicon regions into p5589-mcr-8, thus replacing the original MDR region and generating an additional copy of IS26 and an 8 bp direct repeat (AGCTGCAC) in the fused plasmid pKP09-CTX-M. Subsequently, a 2.3 kb transposition unit containing *bla*_*CTX*-M-15_ was integrated into pKP09-CTX-M through an IS*Ecp1*-mediated transposition event.

### 3.5 Coexistence of plasmid-mediated *bla*_NDM-5_ and *mcr-1.1*

**Figure 4:**
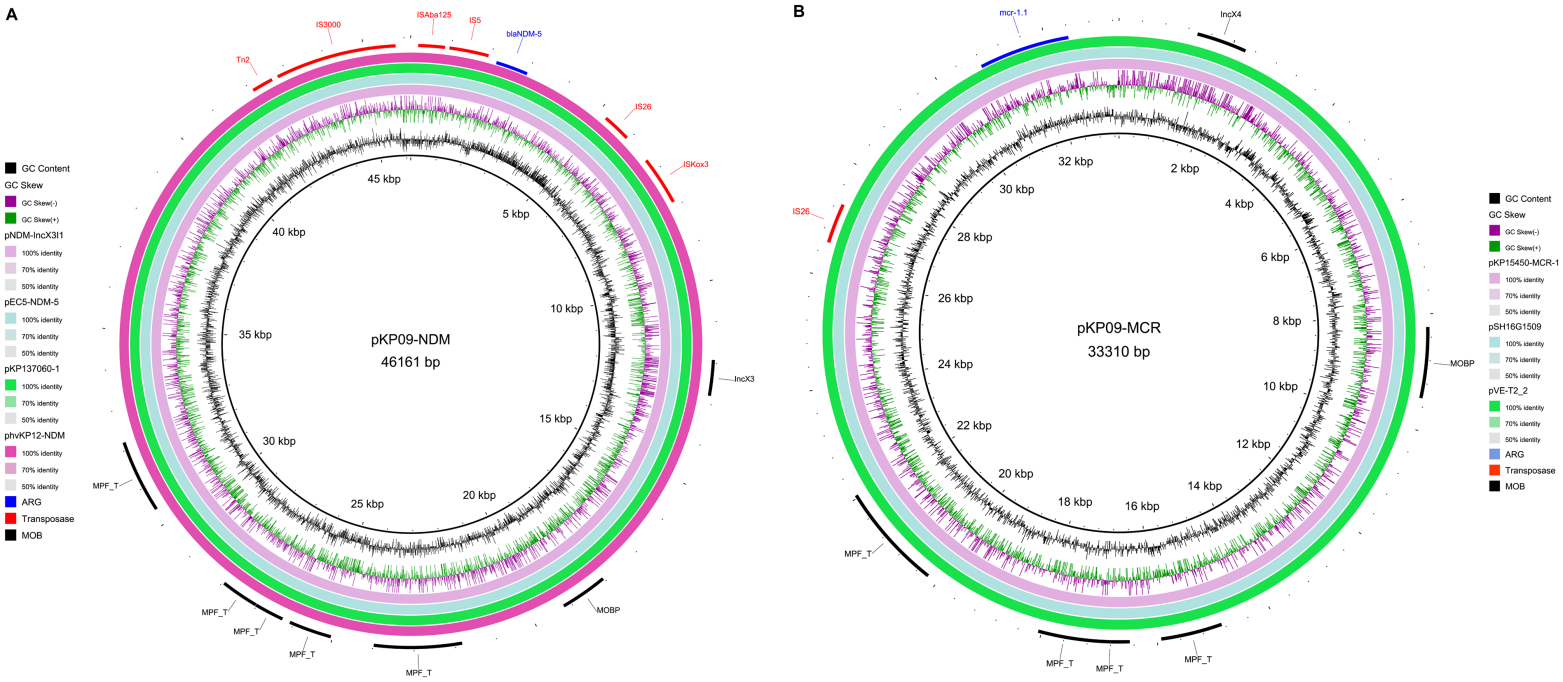
Circular map of two IncX-type plasmids pKP09-NDM carrying *bla*_NDM-5_ **(A)** and pKP09-MCR that carries *mcr-1.1* **(B)**.

We also found that the carbapenemase-encoding gene *bla*_NDM-5_ and the colistin resistance gene *mcr-1.1* were located in two IncX-type plasmids (pKP09-NDM and pKP09-MCR) with a size of 45.1 and 32.5 kb, respectively. The two plasmids were also predicted to be conjugative. In the IncX3-type plasmid pKP09-NDM, *bla*_NDM-5_, and *trpF* with the other two ORFs were flanked by IS*5* upstream and IS*26* downstream. A megaBLAST analysis of this genetic context was highly conserved with 100% sequence identity and coverage between several plasmids, such as pNDM-IncX3I1 and pEC5-NDM-5 in *E. coli*, as well as pKP137060-1 and phvKP12-NDM in *K. pneumoniae* (Figure 4A and Supplementary Table S6).

The core of pKP09-MCR was strikingly similar with (the query cover and identity of 100%) other previously sequenced IncX4-type plasmids that carry *mcr-1*, such as pVE-T2_2 of *E. coli*, pSH16G1509 of *Salmonella enterica*, and pKP15450-MCR-1 of *K. pneumoniae* (Figure 4B and Supplementary Table S6). Furthermore, the *mcr-1.1* gene in pKP09-MCR was carried by the genetic structure of IS*26-parA-mcr-1.1-pap2*. These results showed that IncX4 plasmids harboring *mcr-1* could spread in different species of *Enterobacteriaceae*.

## 4 Discussion

In this study, we obtained an XDR *K. pneumoniae* isolate, KP09, from an intestinal sample of our patient, which exhibited resistance to commonly used antimicrobial agents, including carbapenems, aztreonam, aminoglycosides, tigecycline, colistin, ceftazidime/avibactam, and eravacycline. Eravacycline, as a newly introduced antibiotic into clinical use, has very few reports of resistance (Xu et al., 2022). Fortunately, the patient has not developed an infection at present, but continued monitoring is necessary. Reports have shown that gut-colonizing bacteria are closely linked to subsequent infections, with 24.7% of carbapenem-resistant Enterobacteriaceae (CRE) carriers eventually developing CRE infections (Chu et al., 2022). If an infection occurs, it will become almost untreatable since cefiderocol is not yet available in China. Therefore, it is essential to conduct in-depth studies on the genetic structure and evolutionary characteristics of this strain.

We performed a comprehensive genomic analysis focusing on plasmids that carry multiple ARGs, including *tmexCD1-toprJ1*, *bla*_CTX-M-15_, *bla*_NDM-5_, and *mcr-1.1*. A notable finding was the identification of a novel IncFIA/IncFII/IncQ1 type fusion plasmid that harbors *bla*_CTX-M-15_, which has not previously been reported in the literature. This discovery provides new information on the genetic structure of CTX-M-15-carrying plasmids and their potential role in the spread of extended-spectrum β-lactamase (ESBL) resistance in *K. pneumoniae*.

Previous studies have reported that *bla*_CTX-M-15_ was carried out in various plasmids, particularly IncF and IncI plasmids, which are known to promote the spread of ESBLs among *Enterobacteriaceae* (Yu et al., 2024). A previous study identified a fusion hotspot in conjugative helper plasmids that can be targeted by IS elements like IS*26* and IS*Ecp1* in MDR plasmids, triggering intermolecular transposition and circular intermediate formation, followed by reinsertion into the original nonconjugative plasmid via replicative transposition (Xie et al., 2020). This study identified a new IncFIA/IncFII/IncQ1 hybrid plasmid that contained a *bla*_CTX-M-15_ and an MDR region for 13 other ARGs. The MDR region, originating from an IncFIA/IncQ1/IncR plasmid, recombined with an IncFIA/IncFII plasmid via IS*26*, forming the chimeric plasmid, which then acquired *bla*_CTX-M-15_ through IS*Ecp1*-mediated transposition. The fusion structure we observed in KP09 may offer a more efficient mechanism for the dissemination of CTX-M-15, especially in clinical settings where the rapid spread of resistance is a critical concern. The presence of *bla*_CTX-M-15_ as part of a larger genetic structure may also contribute to the adaptability of *K. pneumoniae* to changing environmental pressures, including the selective use of broad-spectrum β-lactams.

We also observed that the *bla*_NDM-5_ gene and *mcr-1.1* were located on two separate plasmids, IncX3 and IncX4, respectively. These findings are consistent with other studies that have documented widespread dissemination of *bla*_NDM-5_ and *mcr-1* in various Enterobacteriaceae species, including *E. coli* and *K. pneumoniae* (Shen et al., 2018; Kuang et al., 2022). Interestingly, we tested 16 transconjugants selected in meropenem and sodium azide-containing medium, and all were found to carry both pKP09-NDM and pKP09-MCR plasmids simultaneously. Therefore, we speculate that transfer of the IncX3 plasmid that contains the *bla*_NDM-5_ gene in the KP09 strain may require the help of the pKP09-MCR plasmid. The results of the conjugation experiment showed that the pKP09-MCR plasmid had a high transfer frequency, indicating that this plasmid possesses strong transmissibility. From a molecular perspective, both plasmids were predicted to be conjugative, which indicates their potential to transfer between bacterial strains and species, further enhancing the risk of horizontal gene transfer after exposure to colistin (Xiao et al., 2022).

One of the most concerning findings in this study was the presence of *tmexCD1-toprJ1*, a recently identified resistance mechanism that confers resistance to tigecycline and other antibiotics. First reported in China, this efflux pump has been rapidly disseminating in both clinical and agricultural settings (Lv et al., 2020). In our study, we found that the transconjugants carrying *tmexCD1-toprJ1* exhibited an increased MIC (more than 10-fold) to the recently introduced antibiotic eravacycline, suggesting that *tmexCD1-toprJ1* may also be a potential factor contributing to eravacycline resistance in *K. pneumoniae*. The plasmid-encoded *tmexCD1-toprJ1* in KP09 is widely distributed among *Enterobacteriaceae*, including *K. pneumoniae*, *K. oxytoca*, and *E. coli*, highlighting its dissemination and underscoring the growing threat posed by MDR *K. pneumoniae*, especially given the limited treatment options available for infections caused by these strains.

We identified a novel sequence type, ST5491, in KP09, which differs from the endemic ST15 lineage by a single *tonB* allele. Its classification as KL102-O1 and high similarity to other *K. pneumoniae* strains from China and Japan suggest regional dissemination. Phylogenetic analysis further links KP09 to a strain from Beijing, highlighting ongoing genetic diversification. This new ST is concerning as it may represent an emerging lineage associated with the spread of antimicrobial resistance genes (ARGs). Given that certain STs are more prone to acquiring and disseminating resistance (Liu et al., 2014; Peirano et al., 2020), our findings underscore the need for continuous surveillance to track the evolution and resistance potential of *K. pneumoniae*.

## 5 Conclusion

This study provides important information on the genomic structure and resistance mechanisms of an XDR *K. pneumoniae* strain, including the discovery of a new *bla*_CTX-M-15_ gene encoding a IncFIA/IncFII/IncQ1 hybrid plasmid and a new ST. Our findings highlight the urgent need for more effective strategies to combat antimicrobial resistance in *K. pneumoniae*, including stricter antibiotic management, improved surveillance systems, and the development of novel therapeutic agents. Understanding the genetic mechanisms that underlie resistance will be crucial in informing public health strategies to prevent the further spread of multidrug-resistant *K. pneumoniae*.

## Data Availability

The datasets for this study can be found in the online repositories. The genome sequence of the strain has been deposited in the NCBI database under BioProject accession number PRJNA1211817.

## Footnotes

^1^ https://github.com/rrwick/Filtlong

^2^ https://pubmlst.org/

^3^ https://github.com/tseemann/snippy

## References

[ref1] Acman M. Wang R. van Dorp L. Shaw L. P. Wang Q. Luhmann N.. (2022). Role of mobile genetic elements in the global dissemination of the carbapenem resistance gene *bla*_NDM_. Nat. Commun. 13:1131. doi: 10.1038/s41467-022-28819-2, PMID: 35241674 PMC8894482

[ref2] Alikhan N.-F. Petty N. K. Zakour N. L. B. Beatson S. A. (2011). BLAST Ring Image Generator (BRIG): simple prokaryote genome comparisons. BMC Genomics 12:402. doi: 10.1186/1471-2164-12-402, PMID: 21824423 PMC3163573

[ref3] Bengoechea J. A. Sa Pessoa J. (2019). *Klebsiella pneumoniae* infection biology: living to counteract host defences. FEMS Microbiol. Rev. 43, 123–144. doi: 10.1093/femsre/fuy043, PMID: 30452654 PMC6435446

[ref4] Chen S. Zhou Y. Chen Y. Gu J. (2018). fastp: an ultra-fast all-in-one FASTQ preprocessor. Bioinformatics 34, i884–i890. doi: 10.1093/bioinformatics/bty560, PMID: 30423086 PMC6129281

[ref9002] Chu W. Hang X. Li X. Ye N. Tang W. Zhang Y.. (2022). Bloodstream Infections in Patients with Rectal Colonization by Carbapenem-Resistant Enterobacteriaceae: A Prospective Cohort Study. IDR Volume 15, 6051–6063. doi: 10.2147/IDR.S383688 PMC9581720 36277248

[ref5] Croucher N. J. Page A. J. Connor T. R. Delaney A. J. Keane J. A. Bentley S. D.. (2015). Rapid phylogenetic analysis of large samples of recombinant bacterial whole genome sequences using Gubbins. Nucleic Acids Res. 43:e15. doi: 10.1093/nar/gku1196, PMID: 25414349 PMC4330336

[ref6] Feng Y. Zou S. Chen H. Yu Y. Ruan Z. (2021). BacWGSTdb 2.0: a one-stop repository for bacterial whole-genome sequence typing and source tracking. Nucleic Acids Res. 49, D644–D650. doi: 10.1093/nar/gkaa821, PMID: 33010178 PMC7778894

[ref7] Florensa A. F. Kaas R. S. Clausen P. T. L. C. Aytan-Aktug D. Aarestrup F. M. (2022). ResFinder—an open online resource for identification of antimicrobial resistance genes in next-generation sequencing data and prediction of phenotypes from genotypes. Microb. Genom. 8:000748. doi: 10.1099/mgen.0.000748, PMID: 35072601 PMC8914360

[ref8] Gurevich A. Saveliev V. Vyahhi N. Tesler G. (2013). QUAST: quality assessment tool for genome assemblies. Bioinformatics 29, 1072–1075. doi: 10.1093/bioinformatics/btt086, PMID: 23422339 PMC3624806

[ref9] Kolmogorov M. Yuan J. Lin Y. Pevzner P. A. (2019). Assembly of long, error-prone reads using repeat graphs. Nat. Biotechnol. 37, 540–546. doi: 10.1038/s41587-019-0072-8, PMID: 30936562

[ref10] Kuang X. Yang R. Ye X. Sun J. Liao X. Liu Y.. (2022). NDM-5-producing *Escherichia coli* co-harboring *mcr-1* gene in companion animals in China. Animals 12:1310. doi: 10.3390/ani12101310, PMID: 35625156 PMC9137672

[ref11] Lam M. M. C. Wick R. R. Watts S. C. Cerdeira L. T. Wyres K. L. Holt K. E. (2021). A genomic surveillance framework and genotyping tool for *Klebsiella pneumoniae* and its related species complex. Nat. Commun. 12:4188. doi: 10.1038/s41467-021-24448-3, PMID: 34234121 PMC8263825

[ref12] Letunic I. Bork P. (2021). Interactive Tree of Life (iTOL) v5: an online tool for phylogenetic tree display and annotation. Nucleic Acids Res. 49, W293–W296. doi: 10.1093/nar/gkab301, PMID: 33885785 PMC8265157

[ref13] Liu Y. Li X.-Y. Wan L.-G. Jiang W.-Y. Yang J.-H. Li F.-Q. (2014). Virulence and transferability of resistance determinants in a novel *Klebsiella pneumoniae* sequence type 1137 in China. Microb. Drug Resist. 20, 150–155. doi: 10.1089/mdr.2013.0107, PMID: 24236613

[ref14] Liu J.-H. Liu Y.-Y. Shen Y.-B. Yang J. Walsh T. R. Wang Y.. (2024). Plasmid-mediated colistin-resistance genes: mcr. Trends Microbiol. 32, 365–378. doi: 10.1016/j.tim.2023.10.006 38008597

[ref15] Liu B. Zheng D. Zhou S. Chen L. Yang J. (2022). VFDB 2022: a general classification scheme for bacterial virulence factors. Nucleic Acids Res. 50, D912–D917. doi: 10.1093/nar/gkab1107, PMID: 34850947 PMC8728188

[ref16] Lv L. Wan M. Wang C. Gao X. Yang Q. Partridge S. R.. (2020). Emergence of a plasmid-encoded resistance-nodulation-division efflux pump conferring resistance to multiple drugs, including tigecycline, in *Klebsiella pneumoniae*. mBio 11:e02930. doi: 10.1128/mBio.02930-19, PMID: 32127452 PMC7064769

[ref17] Musiał K. Petruńko L. Gmiter D. (2024). Simple approach to bacterial genomes comparison based on average nucleotide identity (ANI) using fastANI and ANIclustermap. Acta Univ. Lodz. Folia Biol. Oecol. 18, 66–71. doi: 10.18778/1730-2366.18.10

[ref18] Navon-Venezia S. Kondratyeva K. Carattoli A. (2017). *Klebsiella pneumoniae*: a major worldwide source and shuttle for antibiotic resistance. FEMS Microbiol. Rev. 41, 252–275. doi: 10.1093/femsre/fux013, PMID: 28521338

[ref19] Néron B. Littner E. Haudiquet M. Perrin A. Cury J. Rocha E. P. (2022). IntegronFinder 2.0: identification and analysis of integrons across bacteria, with a focus on antibiotic resistance in *Klebsiella*. Microorganisms 10:700. doi: 10.3390/microorganisms10040700, PMID: 35456751 PMC9024848

[ref20] Olson R. D. Assaf R. Brettin T. Conrad N. Cucinell C. Davis J. J.. (2023). Introducing the bacterial and viral bioinformatics resource center (BV-BRC): a resource combining PATRIC, IRD and ViPR. Nucleic Acids Res. 51, D678–D689. doi: 10.1093/nar/gkac1003, PMID: 36350631 PMC9825582

[ref21] Overbeek R. Olson R. Pusch G. D. Olsen G. J. Davis J. J. Disz T.. (2014). The SEED and the Rapid Annotation of microbial genomes using Subsystems Technology (RAST). Nucleic Acids Res. 42, D206–D214. doi: 10.1093/nar/gkt1226, PMID: 24293654 PMC3965101

[ref22] Pan F. Wang C. Yang Y. Guo Y. Zhu D. Zhang H.. (2024). Trends in antimicrobial resistance in Enterobacterales isolated from children: data from the China Antimicrobial Surveillance Network (CHINET) from 2015–2021. One Health Adv. 2:21. doi: 10.1186/s44280-024-00054-y

[ref23] Parks D. H. Imelfort M. Skennerton C. T. Hugenholtz P. Tyson G. W. (2015). CheckM: assessing the quality of microbial genomes recovered from isolates, single cells, and metagenomes. Genome Res. 25, 1043–1055. doi: 10.1101/gr.186072.114, PMID: 25977477 PMC4484387

[ref24] Peirano G. Chen L. Kreiswirth B. N. Pitout J. D. D. (2020). Emerging antimicrobial-resistant high-risk *Klebsiella pneumoniae* clones ST307 and ST147. Antimicrob. Agents Chemother. 64:e01148. doi: 10.1128/AAC.01148-20, PMID: 32747358 PMC7508593

[ref25] Puterová J. Martínek T. (2021). digIS: towards detecting distant and putative novel insertion sequence elements in prokaryotic genomes. BMC Bioinformatics 22:258. doi: 10.1186/s12859-021-04177-6, PMID: 34016050 PMC8147514

[ref26] Riwu K. H. P. Effendi M. H. Rantam F. A. (2020). A review of extended spectrum β-lactamase (ESBL) producing *Klebsiella pneumoniae* and multidrug resistant (MDR) on companion animals. System. Rev. Pharm. 11, 270–277. doi: 10.31838/srp.2020.7.43

[ref27] Robertson J. Nash J. H. (2018). MOB-suite: software tools for clustering, reconstruction and typing of plasmids from draft assemblies. Microb. Genom. 4:e000206. doi: 10.1099/mgen.0.000206, PMID: 30052170 PMC6159552

[ref28] Shen Z. Hu Y. Sun Q. Hu F. Zhou H. Shu L.. (2018). Emerging carriage of NDM-5 and MCR-1 in *Escherichia coli* from healthy people in multiple regions in China: a cross sectional observational study. EClinicalMedicine 6, 11–20. doi: 10.1016/j.eclinm.2018.11.003, PMID: 31193653 PMC6537561

[ref29] Sheng Z.-K. Hu F. Wang W. Guo Q. Chen Z. Xu X.. (2014). Mechanisms of tigecycline resistance among *Klebsiella pneumoniae* clinical isolates. Antimicrob. Agents Chemother. 58, 6982–6985. doi: 10.1128/AAC.03808-14, PMID: 25182649 PMC4249433

[ref30] Siguier P. Pérochon J. Lestrade L. Mahillon J. Chandler M. (2006). ISfinder: the reference centre for bacterial insertion sequences. Nucleic Acids Res. 34, D32–D36. doi: 10.1093/nar/gkj014, PMID: 16381877 PMC1347377

[ref31] Smiline A. Vijayashree J. Paramasivam A. (2018). Molecular characterization of plasmid-encoded *blaTEM, blaSHV* and *blaCTX-M* among extended spectrum β*-lactamases* [ESBLs] producing *Acinetobacter baumannii*. Br. J. Biomed. Sci. 75, 200–202. doi: 10.1080/09674845.2018.1492207, PMID: 29962277

[ref32] Stamatakis A. (2014). RAxML version 8: a tool for phylogenetic analysis and post-analysis of large phylogenies. Bioinformatics 30, 1312–1313. doi: 10.1093/bioinformatics/btu033, PMID: 24451623 PMC3998144

[ref33] Wang Y. Cao W. Zhu X. Chen Z. Li L. Zhang B.. (2012). Characterization of a novel *Klebsiella pneumoniae* sequence type 476 carrying both bla KPC-2 and bla IMP-4. Eur. J. Clin. Microbiol. Infect. Dis. 31, 1867–1872. doi: 10.1007/s10096-011-1512-7, PMID: 22271301

[ref34] Wick R. R. Judd L. M. Gorrie C. L. Holt K. E. (2017). Unicycler: resolving bacterial genome assemblies from short and long sequencing reads. PLoS Comput. Biol. 13:e1005595. doi: 10.1371/journal.pcbi.1005595, PMID: 28594827 PMC5481147

[ref35] Xiao X. Zeng F. Li R. Liu Y. Wang Z. (2022). Subinhibitory concentration of colistin promotes the conjugation frequencies of *Mcr-1*- and *bla*_NDM-5_-positive plasmids. Microbiol. Spectr. 10:e02160. doi: 10.1128/spectrum.02160-21, PMID: 35230128 PMC9045390

[ref36] Xie M. Chen K. Ye L. Yang X. Xu Q. Yang C.. (2020). Conjugation of virulence plasmid in clinical *Klebsiella pneumoniae* strains through formation of a fusion plasmid. Adv. Biosyst. 4:e1900239. doi: 10.1002/adbi.201900239, PMID: 32293159

[ref9001] Xu C. Wei X. Jin Y. Bai F. Cheng Z. Chen S.. (2022). Development of Resistance to Eravacycline by Klebsiella pneumoniae and Collateral Sensitivity-Guided Design of Combination Therapies. Microbiol Spectr 10, e01390–22. doi: 10.1128/spectrum.01390-22 35972286 PMC9603973

[ref37] Yu K. Huang Z. Xiao Y. Gao H. Bai X. Wang D. (2024). Global spread characteristics of CTX-M-type extended-spectrum β-lactamases: a genomic epidemiology analysis. Drug Resist. Updat. 73:101036. doi: 10.1016/j.drup.2023.101036, PMID: 38183874

