# Supplementary figures and images for: Emergence of an XDR Klebsiella pneumoniae ST5491 strain co-harboring NDM-5, MCR-1.1, tmexCD1-toprJ1, and a novel plasmid carrying CTX-M-15
Source: Front Microbiol. 2025 Apr 30;16:1581851. doi: 10.3389/fmicb.2025.1581851 (PMC12075367; doi:10.3389/fmicb.2025.1581851)

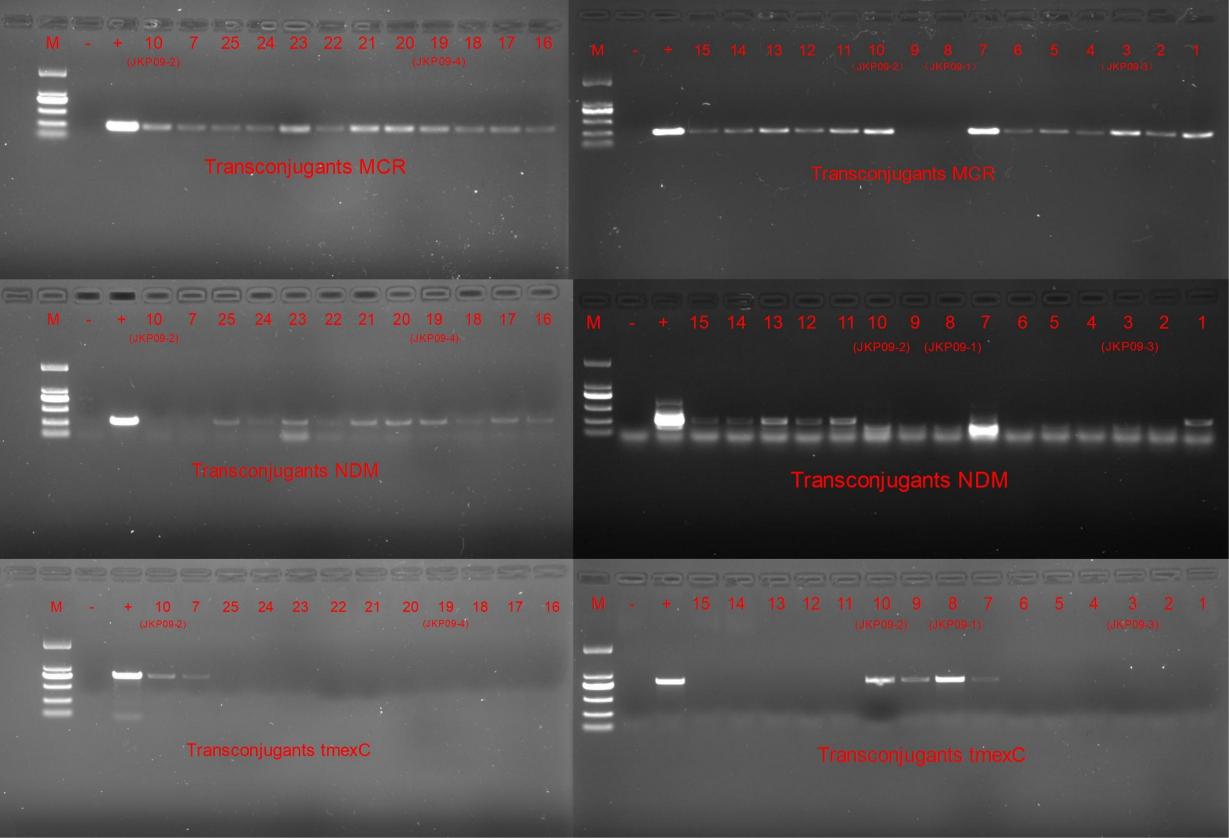


**Supplementary Figure S1. PCR results of the transconjugants.**

Supplement: Supplementary file 1 [file Data_Sheet_1.docx]
