# Supplementary material for: Emergence of an XDR Klebsiella pneumoniae ST5491 strain co-harboring NDM-5, MCR-1.1, tmexCD1-toprJ1, and a novel plasmid carrying CTX-M-15
Source: Front Microbiol. 2025 Apr 30;16:1581851. doi: 10.3389/fmicb.2025.1581851 (PMC12075367; doi:10.3389/fmicb.2025.1581851)
Supplement: Supplementary file 2 [file Data_Sheet_2.docx]

**Supplementary Figure S2. Comparative schematic of five small plasmids from the KP09 genome (A: pKP09-5, B: pKP09-6, C: pKP09-7, D: pKP09-8, E: pKP09-9) with their two highly homologous counterparts (sequence coverage >95% and identity >99.9%).** The outer rings depict aligned sequences between the homologous plasmids and corresponding KP09 plasmids, labeled with their NCBI Assembly Accession numbers.
